# Multiscale Modeling of Vinyl-Addition Polynorbornenes: The Effect of Stereochemistry

**DOI:** 10.3390/polym16162243

**Published:** 2024-08-07

**Authors:** Nobahar Shahidi, Jeffrey A. Laub, Konstantinos D. Vogiatzis, Manolis Doxastakis

**Affiliations:** 1 Department of Chemical and Biomolecular Engineering, University of Tennessee, Knoxville, TN 37996, USA; 2 Department of Chemistry, University of Tennessee, Knoxville, TN 37996, USA

**Keywords:** molecular dynamics, polynorbornenes, coarse-grained, helical polymer

## Abstract

Vinyl-addition polynorbornenes are candidates for designing high-performance polymers due to unique characteristics, which include a high glass transition temperature associated with a rigid backbone. Recent studies have established that the processability and properties of these polymers can be fine-tuned by using targeted substitutions. However, synthesis with different catalysts results in materials with distinct properties, potentially due to the presence of various stereoisomers that are difficult to quantify experimentally. Herein, we develop all-atom models of polynorbornene oligomers based on classical force fields and density functional theory. To establish the relationship between chemical architecture, chain conformations, and melt structure, we perform detailed molecular dynamics simulations with the fine-tuned atomistic force field and propose simpler coarse-grained descriptions to address the high molecular weight limit. All-atom simulations of oligomers suggest high glass transition temperatures in the range of 550–600 K. In the melt state (800 K), meso chains form highly rigid extended coils ($C_{\infty }\approx 11$) with amorphous structural characteristics similar to the X-ray diffraction data observed in the literature. In contrast, simulations with racemo chains predict highly helical tubular chain conformations that could promote assembly into crystalline structures.

## 1. Introduction

Vinyl-addition polynorbornenes (PNBs) display unique characteristics such as a high glass transition temperature ($T_{g}$), high thermal and chemical stability, low dielectric constant, non-hygroscopicity, high transparency, low birefringence, and high permeability [1,2,3]. Therefore, they could be used in thermoplastic elastomers [4,5], high-performance polymers for gas separation membranes [6,7,8,9,10,11,12,13], microelectronic applications [14,15], as well as anion-exchange membranes and fuel cells [16,17,18,19,20,21]. In several of these applications, selective functional substitutions are incorporated to increase melt processability and tailor properties to the desired specifications [5,8,10,11,12,14,22,23,24,25,26,27].

**Figure 1:**
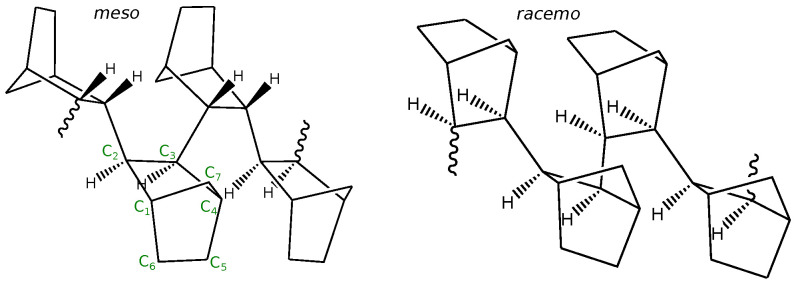
Stereoregular isomers of PNB with 2,3 polymerization: erythro-diisotactic (meso) and erythro-disyndiotactic (racemo). Only the backbone hydrogens are drawn to denote the product of *exo*-polymerization. Carbon atoms are labeled for one selected ring. Note, the different arrangement of bridging carbons (C_7_) for successive rings. Rotatable C_2_-C_3_-C_2_-C_3_ backbone dihedrals are shown in the *trans* conformation.

Unsubstituted PNBs are not melt processable due to the small window (∼30 K) between their $T_{g}$ (493–663 K) [14,28,29] and thermal decomposition temperature [5]. PNBs are also brittle and in most cases exhibit poor solubility in common organic solvents [28]. However, such properties vary significantly depending on synthesis conditions, such as the catalyst used in the polymerization reaction [30,31]. Therefore, soluble unsubstituted PNBs have been reported in the literature [28,32,33,34,35]. This discrepancy has been attributed to the presence of distinct isomers, which is supported by the stereo-specificity of different metallocene precursors [36,37]. While six different stereoregular norbornene polymers could be formed, the most common *cis-exo* insertion is expected to lead to erythro-diisotactic (meso), erythro-disyndiotactic (racemic) or erythro-atactic sequences (random) with stereoregular tetrades for the first two illustrated in Figure 1 [1,36]. Another reported feature that depends on the employed catalyst is the formation of C7 linkages that could render the material insoluble [38,39,40]. The structure-property relationship of these isomeric sequences has long been investigated with theory, experiments, and simulations to overcome the challenges of processing and characterizing these polymeric materials.

Haselwander et al. [28] reported vinyl addition polymerization of norbornene with Pd^2+^ complexes that produce amorphous, high-$T_{g}$, soluble polymers with a characteristic ratio ($C_{\infty }$) of 11.4 representative of stiff random coils. To gain further insight, they performed ab initio AM1 and STO-3G rotational potential calculations around the bond linking the monomers of a racemo dimer. The rotational isomeric state (RIS) model of a chain with ∼160 monomers produced $C_{\infty }=12.1$ and short (450 ps) simulations of single chains with 100 monomers using the Dreiding force field [41] resulted in a lower value of $C_{\infty }$ = 8.5 [42] which could be attributed to the consideration of higher-order dihedral correlations absent in the earlier RIS calculations or to chain contraction due to the poor model solvent (vacuum). Two-dimensional rotational potentials using successive rotatable bonds and dihedral distributions were calculated, suggesting that long-range dihedral correlations can be important for PNBs. Furthermore, simulations by Haselwander et al. [42] emphasized the need for a high degree of polymerization (*N*) to reach polymer-like behavior free of chain-end effects. They also recorded rotational jumps at temperatures higher than 800 K, turning to free rotations at 1600 K. Similar results were also reported with nanosecond-long trajectories at 600 K in the condensed phase; quenching such systems produced a change in the specific volume at 478 K, suggesting the onset of $T_{g}$ [43].

Ahmed et al. [44] derived all-atom (AA) force fields for PNB by using ab initio calculations and semi-empirical methods with model dimers to characterize bond, angle bending, and rotational potentials separately for meso and racemo PNBs. They adopted intermolecular interactions with Lennard–Jones parameters from the Dreiding force field. Subsequent work by Wilks et al. [27] proposed that the angle force constants were overestimated and opted for standard CHARMM parameters [45]. The extracted rotational potentials were implemented in Monte Carlo simulations of single chains in vacuum for meso, racemo, and 50/50 atactic oligomers. They reported that the meso chain resembles a rigid rod, and the racemo is semi-rigid, while the conformation of the atactic chain appears closer to a random coil. These data were qualitatively compared to the experimental intrinsic viscosities of PNB and its alkane-substituted derivatives synthesized with different catalysts. They proposed that the intractable polymers produced by Pd catalysts have a high ratio of meso sequences (assuming no impact of hydrocarbon side chains on polymer conformation) [37,46]. Additional RIS calculations with single chains consisting of the meso isomer predicted a helix with successive dihedrals of 120 or 240°. This model oligomer was close to a rigid rod; however, they theorized that kinks would appear in sufficiently long chains and produce a random coil conformation at high molecular weights. The presence of a certain degree of helicity led to the hypothesis that PNB with this stereochemistry could present a degree of ordering, but the prevalence of kinks would impede its crystallization [46,47].

The condensed phase structure of PNBs has also been investigated extensively with wide-angle X-ray diffraction. Soluble PNBs display an amorphous structure with two broad peaks in the wide-angle regime [28,48,49,50]. Ahmed et al. reproduced the two peaks with amorphous chains of meso PNB, discussing that the most dominant contributions to the first peak originated from intermolecular correlations, while the second is mostly the result of intrachain structure [46]. Experiments and short molecular dynamics (MD) simulations with substituted PNBs further support this assignment, with functionalization systematically impacting the first peak while the location of the second peak remains intact [27]. Kai et al. examined PNBs synthesized with Pd and Ni catalysts and reported significant differences between their properties, especially for unsubstituted PNBs. Pd catalysts lead to higher interchain ordering, as evidenced by the first scattering peak and viscosity measurements. This was postulated to be due to higher stereoregularity and fewer kinks in the rod-like conformation [50].

In addition to amorphous structures, Porri et al. reported that racemo heptamers form a crystalline structure [51] and Ricci et al. reported that 2,3-*exo*-diheterotactic stereochemistry (random meso-racemo) can result in semicrystalline PNBs [52]. More recently, Buono et al. presented evidence that PNB oligomers can form a crystalline phase. They postulated that similar to Porri [51], their oligomers had a racemo microstructure. Their simulations showed that the lowest energy for racemo was a six-fold helical conformation [53].

In this study, we used MD simulations to directly correlate bulk properties with PNB backbone stereochemistry. First, we employed extensive density functional theory (DFT) calculations of trimers to critically examine the ability of generic AA force fields to represent the strained backbone conformations of PNBs. We found that the Lennard–Jones functional form overestimates short-range repulsion between adjacent repeat units and chose the Williams7B force field [54] instead, which employs the Buckingham non-bonded potential. After optimizing the Williams7B dihedral parameters to match DFT results, we performed melt simulations of PNBs. We also propose a simpler coarse-grained (CG) model that can overcome the molecular weight limitations of AA modeling.

Our results indicate that meso chains form extended and highly rigid coils with amorphous structural characteristics in accordance with X-ray diffraction data in the literature. In contrast, our simulations of racemo chains predict highly helical tubular conformations that could promote crystallization.

## 2. Materials and Methods

### 2.1. Density Functional Theory

DFT calculations were performed for norbornene dimers and trimers. All DFT calculations were performed using the TURBOMOLE 7.2 program package [55] with the PBE0 density functional [56], the def2-TZVPP basis set [57], Grimme’s D3 dispersion correction [58], with the Becke–Johnson damping function [59], and the resolution of identity for the two-electron integrals. Domain-localized pair natural orbital coupled-cluster singles-and-doubles with perturbative triples (DLPNO-CCSD(T)) [60,61] calculations were performed for norbornene dimers using the ORCA 5.0 package [62]. The aug-cc-pVTZ basis set [63,64], was used in all DLPNO-CCSD(T) calculations while the T_CutPNO_ threshold was set to 10^−8^. The resolution of identity approximation for Coulomb and HF exchange was applied together with the aug-cc-pVQZ/C auxiliary basis set.

A benchmark study on norbornene dimer structures was used to validate the choice of the density functional used for the larger trimer units. A potential energy surface was computed along the torsion angle for the meso and the racemo dimer conformations. DLPNO-CCSD(T)/aug-cc-pVTZ calculations on the structures optimized with PBE0-D3(BJ)/def2-TZVPP were used as references. The accuracy of the following density functionals was evaluated: PBE0 [65]. B3LYP [66]. BLYP [67], BP86 [68,69], M06 [70], M06-L [71], PBE [72], and TPSSh [73]. In addition, we performed DLPNO-CCSD(T) calculations with a double-zeta quality basis set (aug-cc-pVDZ), and the error introduced by the smaller basis set size was evaluated with respect to the DLPNO-CCSD(T)/aug-cc-pVTZ energies.

Two successive C_2_-C_3_-C_2_-C_3_ dihedral angles ($\varphi_{1}$ and $\varphi_{2}$) were used to generate the three-dimensional potential energy surfaces of the norbornene trimer structures which have exo-meso-meso, exo-meso-racemo, and exo-racemo-racemo stereochemistry. We have considered molecular geometries where $\varphi_{1}$ and $\varphi_{2}$ angles varied from −180° to 180° in intervals of 20°. Overall, a total of 324 different structures were generated per trimer. For computing the DFT energy of each structure, the dihedral angles were kept fixed while all the other degrees of freedom were allowed to relax during the geometry optimizations.

### 2.2. Molecular Dynamics Simulations

Simulations were performed with the GROMACS package [74] (version 2019.6). Melt properties were calculated using the Williams7B force field with modified backbone dihedrals, as described in detail in Section 3.1. The original Williams7B parameters, along with the newly extracted set of dihedral coefficients are displayed in Table 1.
**Table 1:** Parameters for Williams7B [54] force field with bonded terms originally adopted from CHARMM force field [75] and dihedrals from Tu et al. [76]. Meso and racemo backbone dihedrals (C_2_-C_3_-C_2_-C_3_) were further optimized for PNBs (this work).

$V_{\mathrm{nb}}\left(r\right)=A\exp\left(-Br\right)-C/r^{6}$ (non-bonded)						
	*A* (kJ/mol)		*B* (nm^−1^)		*C* (nm^6^·kJ/mol)	
C-C	$3.4186\times 10^{5}$		36.94		$2.005\times 10^{-3}$	
C-H	$0.5814\times 10^{5}$		37.61		$0.507\times 10^{-3}$	
H-H	$0.1365\times 10^{5}$		38.30		$0.128\times 10^{-3}$	
$V_{b}\left(b\right)=\frac{1}{2}k_{b}{\left(b-b_{0}\right)}^{2}$ (bond-stretching)						
	$b_{0}$ (nm)			$k_{b}$ (nm^2^·kJ/mol)		
C-C	0.153			224,262.4		
C-H	0.104			284,512.0		
$V_{a}\left(\theta \right)=\frac{1}{2}k_{\theta }{\left(\theta -\theta_{0}\right)}^{2}$ (angle-bending)						
	$\theta_{0}$ (deg.)			$k_{\theta }$ (rad^2^·kJ/mol)		
C-C-C	113.7			488.273		
C-C-H	109.2			313.800		
H-C-H	106.0			276.144		
$V_{d}\left(\phi \right)=\sum_{n=1}^{3}k_{n}\left(1+\cos(n\phi -\phi_{n})\right)$ (dihedral rotation)						
	$k_{1}$ (kJ/mol)	$k_{2}$	$k_{3}$	$\varphi_{1}$ (deg.)	$\varphi_{2}$	$\varphi_{3}$
C-C-C-C	0.9791	0.4017	1.7154	0	0	0
C-C-C-H	0	0	0.7406	0	0	0
H-C-C-H	0	0	0.6904	0	0	0
C2-C3-C2-C3 (m)	−1.3072	0.9035	−3.2567	−159.0	8.6	−4.3
C2-C3-C2-C3 (r)	3.2241	−1.2080	−2.2806	−0.9	180.0	1.0

Melt PNB systems studied with AA models include four different degrees of polymerization (*N*): 10, 22, 30, and 41 repeat units per chain with progressively larger systems for higher *N* to avoid finite-size effects (number of chains *n* = 25, 40, 50, and 55). Four compositions were examined for each *N*, with chains containing 0% (meso system), 50%, 75%, and 100% racemo dimers denoted below as meso, r50, r75, and racemo. In the atactic systems, each chain was constructed with a specific ratio of meso and racemo dimers placed in random sequences.

Chains were initially constructed with a rod-like conformation and relaxed at high temperatures (1000–1300 K). While chemical connectivity is preserved in our AA models, we observe that a small number of backbone hydrogens converted to the *endo*-configuration (see Figure 1) at high temperatures, and we examined our trajectories to verify that the *exo*-fraction remained the prevalent form (above 90%). The majority of simulations were performed at T=800 K, a sufficiently high temperature for the PNB chains to be mobile in the melt state. Select systems were quenched to lower temperatures (with the rate of 2 K/ns) to examine any accessible temperature-induced transitions and compared to experimental data available at low temperatures. Configurations at room temperature were then selected for short simulations (5 ns) used to generate X-ray diffraction data.

We employed a cut-off of 1 nm for the van der Waals interactions of the Williams7B force field with energy and pressure dispersion corrections. Bonds with hydrogen atoms were constrained using the Lincs algorithm [77], which allowed the use of a timestep of 1 fs. Production runs were performed in the canonical (*NVT*) ensemble using the velocity re-scaling thermostat [78]. Densities were calculated from isothermal–isobaric (*NPT*) simulations using the Parrinello–Rahman barostat [79], while annealing and equilibration *NPT* runs were performed with the Berendsen barostat [80]. Data for PNB-N41 systems at 800 K were extracted from 200 ns *NVT* simulations and 20 ns *NPT* simulations.

Based on the size and orientation correlation of the modeled chains, such extended simulation times are necessary to generate equilibrated structures despite the high temperature. To expand our studies to higher *N*, we derived CG models, as described further in Section 3.1. MD simulations with CG representation were performed with the same software and methods, although a higher timestep (4 fs) was applicable without sacrificing stability in our computational experiments.

Additional calculations with AA models were performed with dimers and trimers in a vacuum to compare with DFT. Energy minimization was performed with double-precision GROMACS using the steepest descent algorithm and convergence tolerance of 1 kJ mol^−1^nm^−1^. Finally, for comparison, we also performed a limited set of simulations of melt systems with the OPLS-AA force field [81], using a cut-off of 1.5 nm.

## 3. Results

### 3.1. Force Field Optimization

**Figure 2:**
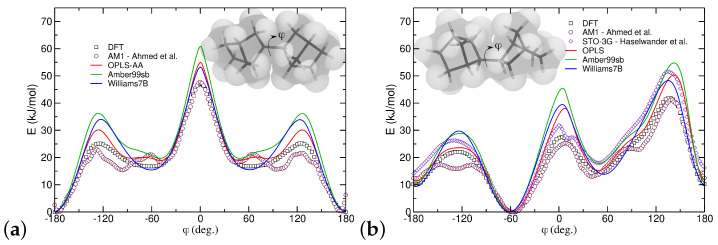
Minimum energy of dimers as a function of the backbone dihedral ϕ (C2-C3-C2-C3) for (**a**) meso and (**b**) racemo isomers. Results from DFT and MD simulations using the OPLS-AA [81], Amber99sb [85], and Williams7B [54] force fields, alongside data from literature using the AM1 semiempirical method [44] and STO-3G ab initio quantum mechanical calculations [28].

PNBs have a highly strained bicyclic backbone with a restricted rotation that can be challenging to model with generic force fields [28,44,82]. Studies of the secondary structure of polypeptides have shown that such force fields generally do not reproduce experimental results regarding helicity [83] but can be improved by optimization of backbone dihedral potentials [84]. Herein, we evaluated several classical force fields in terms of describing minimal energies of dimer structures for specific dihedral values of the main backbone torsion (C_2_-C_3_-C_2_-C_3_) with the highest degree of freedom and contrasted these results to our own DFT calculations as well as past data extracted with semiempirical methods. Overall, our DFT results are in good agreement with semiempirical calculations for the two different stereoisomers, as shown in Figure 2.

The energy barriers of backbone rotation were extracted using several generic AA force fields, including OPLS-AA [81], Amber99sb [85], and Williams7B [54], and are significantly higher than DFT calculations as shown in Figure 2. Previous studies reported similar findings when comparing the Dreiding force field to semiempirical methods [44]. We attribute this discrepancy to the steep repulsion between hydrogen atoms in successive rings; for specific values of ϕ, their distances are below the crossover from repulsive to attractive non-bonded energy in Lennard–Jones potentials. The proximity of the ring hydrogen atoms is qualitatively illustrated in the insets of Figure 2.

**Figure 3:**
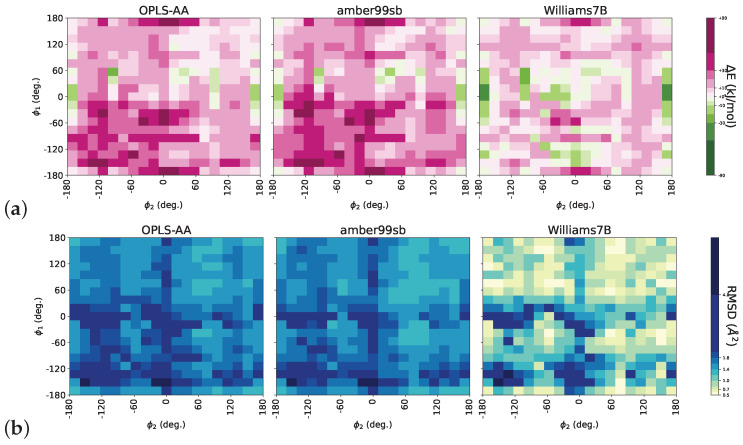
(**a**) Energy difference (ΔE) between minimal energies of classical force fields relative to DFT for a meso-racemo trimer as a function of two successive backbone C_2_-C_3_-C_2_-C_3_ dihedrals, $\phi_{1}$ and $\phi_{2}$. Left-to-right: OPLS-AA [81], Amber99sb [85], and Williams7B [54]. (**b**) Root mean square deviation (RMSD) between structures after energy minimization with generic classical force fields and DFT.

To accommodate for the overestimation of rotational barriers in classical force fields, past studies tuned dihedral potentials based on such dimer calculations [44]. However, simulations with classical force fields have shown that successive C_2_-C_3_-C_2_-C_3_ dihedrals can remain highly correlated along the backbone [42]; therefore, the ability of generic interaction parameters to capture complex intramolecular correlations is critical. In our study, we sought improvements with high-accuracy DFT calculations using trimer sequences. We proceeded with an extensive calculation of minimal energies in three consecutive repeat units as a function of their stereochemistry and the values of two successive backbone (C_2_-C_3_-C_2_-C_3_) dihedrals ($\phi_{1}$, $\phi_{2}$). Several combinations of $\phi_{1}$ and $\phi_{2}$ can lead to major steric overlaps between hydrogen atoms of the first and third repeat units, resulting in high energy barriers. Figure 3a) presents the difference between energies calculated with DFT and three different sets of interaction parameters for one specific trimer (meso-racemo). The OPLS-AA and Amber99sb force fields severely overestimate the energy, especially for highly strained configurations of trimers. These force fields include the common 12-6 Lennard–Jones nonbonded potential, which is known to be too repulsive at short distances [86,87]. In contrast, Williams7 [88] and Williams7B force fields that use the exp-6 Buckingham potential (reported to provide a more accurate repulsive interaction [54,89,90,91,92] at the expense of computational efficiency) best reproduced the features observed with DFT. It is important to recognize that such differences in energies result in structural deviations as well. Figure 3b supports that for a specific pair of ($\phi_{1}$, $\phi_{2}$) and input optimal coordinates from DFT, subsequent minimization using the Williams7B interaction potentials results in the lowest root-mean-squared deviation from DFT structures.

**Figure 4:**
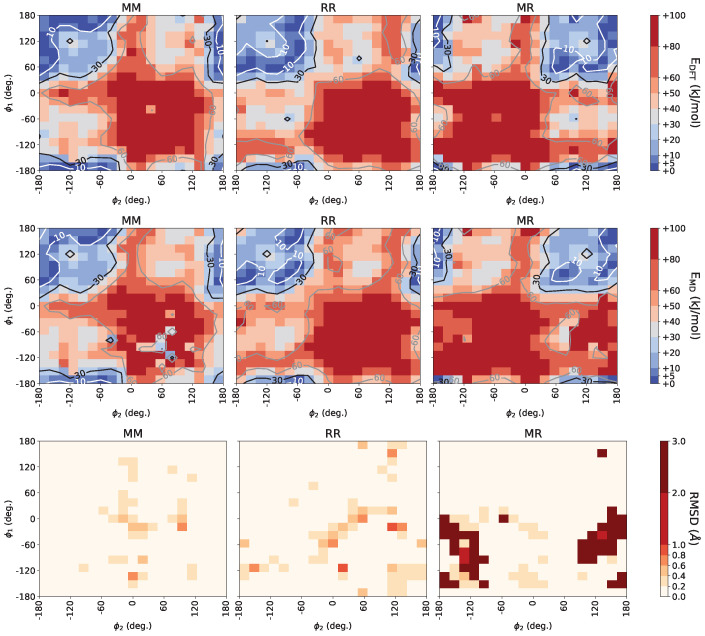
Rotational energy maps of trimers from DFT calculations compared with force field simulations using the Williams7B [54] model with optimized backbone dihedrals for the meso-meso, racemo-racemo, and meso-racemo tacticities. The bottom row displays the RMSD of minimal energy structures relative to initial configurations optimized with DFT.

Motivated by the previous results and data supporting higher accuracy of the force field for predicting the density of oligomers [54], we adopted the updated Williams7B force field for the majority of our study with the exception of limited additional data extracted based on simulations with the OPLS-AA force field. Given our extensive first principle calculations, we performed additional optimization of the meso and racemo backbone dihedral potentials to further improve the predictions of the Williams7B set compared to DFT. The top panel in Figure 4 presents energy maps for all three combinations as a function of $\phi_{1}$ and $\phi_{2}$ extracted from DFT calculations. Each trimer includes two C_2_-C_3_-C_2_-C_3_ which are formed by a meso-meso, racemo-racemo, or meso-racemo sequence. By modifying the dihedral potential for C_2_-C_3_-C_2_-C_3_ as listed in Table 1, minimal energies of trimers from the classical force field simulations capture most features of the DFT energy maps as shown in the second row in Figure 4. A reasonable description for the meso-racemo trimer is achieved as well, although some deviations at high-energy regions persist. All isomer maps present a high-energy region around the zero-zero dihedral conformation and have minimum energies at the trans-trans conformation. The meso-racemo trimer shows significantly lower energies with various high-probability conformations, while the racemo-racemo tacticity has the lowest density of low-energy conformations. Ultimately, the accuracy of the force fields discussed should be corroborated with models of macromolecules in condensed phases. To that extent, a bottom-up multiscale approach was followed below.

### 3.2. Thermodynamic and Structural Properties

**Figure 5:**
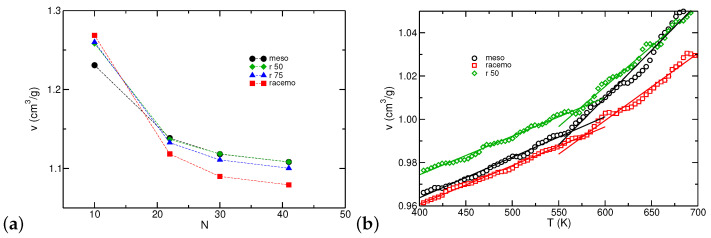
(**a**) Specific volume (*v*) of PNB as a function of chain length for AA simulations at 800 K. (**b**) Specific volume (*v*) as a function of temperature using a cooling rate of 2 K/ns for PNB-N30. Each point represents the average over a 1 K range (5000 points). Solid lines mark linear regressions in the melt and glass regimes intersecting at $T\approx T_{g}$.

As discussed in the methods section, PNB simulations of oligomers at elevated temperatures (>1000 K) displayed rapid dynamics and sufficient conformational transitions to relax our initial structures. At 800 K, systems presented liquid-like densities with dynamics decelerating significantly in agreement with the observations by Haselwander of racemo chains in a vacuum [42]. Figure 5 presents the specific volume *v* at the melt state for all degrees of polymerization *N* studied with AA simulations. At higher chain lengths, *v* decreases following the reduction in chain-end effects [93] with the most dramatic change appearing for racemo chains. Notice that the 100% racemo systems for N>10 appear more densely packed, which suggests higher cohesive energies. We attribute this to changes in single chain structure when exceeding N>10 as discussed further in the next section. We observed that a small fraction of meso dyads suffices to elevate the specific volume to values similar to the pure meso chains.

At T=800 K, equilibration of N=41 required long molecular dynamics trajectories (end-to-end vector relaxation times longer than ≈100 ns) that are computationally expensive (particularly given the force field employed relative to standard Lennard–Jones interactions). Therefore, the direct study of higher *N* values or lower temperatures with AA models is impractical since trajectories commonly explore configurations similar to their initial point. It is important, though, to examine whether the structures probed are in agreement with the experimental data recorded at room temperature. We proceeded by quenching our shortest model systems, using a cooling rate of 2 K/ns starting from the relaxed configurations at 800 K. In principle, such virtual dilatometry experiments could probe temperature-induced transitions. However, the excessively high rates implemented in simulations compared to experimental procedures limit our findings. As an example, $T_{g}$ can be extracted by changes in the thermal expansion coefficient, which, unfortunately, are weak in the case of PNBs as reported by ellipsometry measurements [5]. Computational modeling commonly overestimates $T_{g}$ due to the excessively high cooling rates imposed, although such effects can be counterbalanced by a depression expected when modeling oligomers [94,95,96,97]. Representative results with our systems are shown in Figure 5 with several intriguing findings. First, while all samples exhibit a change in the thermal expansion coefficients at high temperatures, the meso configuration appears to have the lowest point of thermal arrest. The sample with 50% racemo dyads, while starting from the same specific volume as the meso sample, appears to undergo a transition at 20 K higher, in line with observations for the racemo sample. Potential heterogeneities formed by interactions between racemo sequences present in this sample are responsible for this behavior. As a result, the 50% racemo configuration displays a higher specific volume at room temperature compared to the meso configuration. The observed $T_{g}$ values are all in the range of 550–600 K in agreement with the reported value of 658 K [14] particularly when considering any depression due to the oligomeric composition of our samples. The obtained densities for PNB-N22 at 300 K are ∼1.04 g/cm^3^ for all tacticities which is in agreement with the reported value of 1.065 g/cm^3^ [12]. Some experiments reported crystallization for short oligomers with racemo tacticity [53] (as well as syndiotactic meso-racemo [52]), which we did not observe, possibly due to the high cooling rate used in simulations. Further discussion is provided at the end of the Results section.

**Figure 6:**
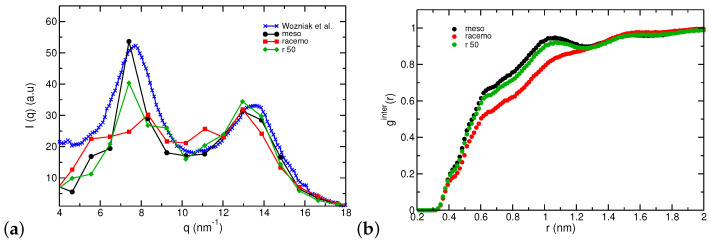
(**a**) X-ray scattering patterns calculated for PNB-N22 at 300 K (from the cooling ramp of 10 K/ns starting from 800 K) compared with WAXS experimental data with unknown tacticity from Wozniak et al. [12] (λ=0.154 nm). (**b**) Inter-molecular radial distribution function based on pairs of carbon atoms (g_CC_).

We compare simulated structures directly to X-ray diffraction data for samples at room temperature as presented in Figure 6a. The positions of the peaks predicted by our model are in good agreement with experimental data [12], which exhibited an amorphous structure. All PNB tacticities have similar peak positions, with high-racemo content resulting in broader signals and high meso content resulting in higher intensity in the first peak. We also examined data from longer molecules (N=41) at 800 K; meso chains exhibited a single peak, while racemo systems appear to display a split first peak at 5 and 8 nm^−1^. We attribute this to the tubular structure long racemo chains display, as will be discussed in the next section, but we refrained from targeting room temperatures directly with higher molecular weights. To expand our analysis on condensed structures, we calculated explicitly the intermolecular radial distribution between carbon atom pairs by subtracting contributions of unwrapped chains (intra) from the total [98]. Figure 6b shows that the position of the neighbor shells is similar across different tacticities. The racemo system has a higher number of local intramolecular neighbors and a longer range to reach melt density in contrast to systems with meso sequences where a very clear intermolecular peak is readily observed at ≈1 nm. Our data suggest that the meso sample (di-isotactic) is closer to experimental data of processable PNBs in agreement with arguments by Wilks et al. and the discussion by Kai et al. [27,50] regarding the first peak’s assignment to intermolecular correlations. However, our meso-samples do not necessarily conform to helical structures, and our density calculations resulted in more compact structures (higher density) for racemo chains; we will discuss such findings further in the following sections.

### 3.3. Chain Conformations

**Figure 7:**
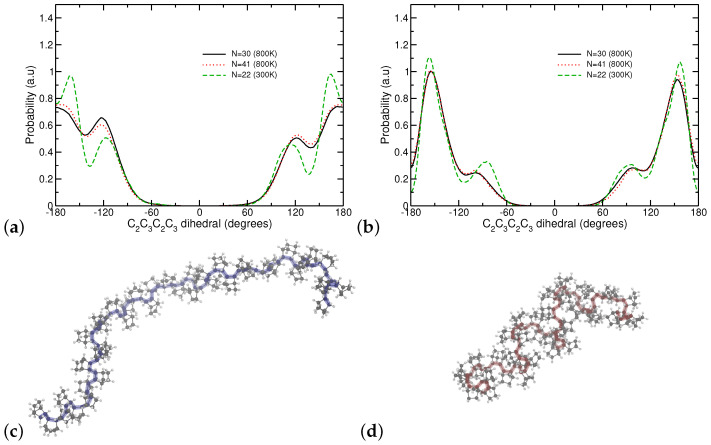
Populations of C_2_-C_3_-C_2_-C_3_ dihedrals probed by simulations with the (**a**) Meso and the (**b**) Racemo stereochemistry with different conditions (degree of polymerization and temperature). Visual representations of a meso (blue backbone) and racemo (red backbone) chain with N=41 at T=800 K are presented in (**c**) and (**d**), respectively.

PNB chains in our systems adopted distinct conformations based on their stereochemistry. We first examined the population characteristics of the rotatable bond between two repeat units characterized by the dihedral C_2_-C_3_-C_2_-C_3_. Figure 7a presents the distribution of this dihedral for the meso architecture. Three different populations are readily identified at trans and ±120° (*skew*). While the peaks at ±120° are in agreement with past RIS calculations [47] our simulations predict a significant affinity for the trans configuration irrespective of *N* and chain ends (compare N=30 to N=22). To examine correlations between successive dihedrals, angles in each chain were probed individually; helical segments consisting of successive identical values of −120° or +120° were uncommon. At 800 K, such segments can rarely extend over 10 repeat units; however, on average, our meso systems displayed about 12% helicity. Reducing the temperature resulted in a higher fraction of *trans* dihedrals that diminished further any helical segments to 5–6%. At low temperatures, a systematic small deviation for trans configurations from −180/180° values is apparent and we attribute such observations to repulsion between hydrogens in neighboring repeat units. Therefore, we conclude that while our models for meso chains present an amorphous structure that is in agreement with X-ray diffraction data, they do not display any significant helicity as was proposed in past studies [46,47].

Our findings for the racemo stereochemistry are in sharp contrast to the meso. While four populations are still observed in Figure 7b, correlations between successive repeat units are dominant. We observed successive dihedrals alternating between positive and negative values of 153° (*transoid*) forming short oligomeric right-handed and left-handed helices. At 800 K, approximately 50% of the repeat units in the racemo sample form such helical segments. To the extent we can probe any changes with our quenching protocol, this fraction remains practically unchanged at 300 K. Porri et al. reported crystal structures of racemo heptamers with distorted *transoid*, *skew*, and *gauche* conformations, and more recently Buono et al. argued that data from X-ray experiments on crystal structures support that polymers form tubular helical segments comprised of sequences of 169° and −129° and a period of six repeat units [51,53]. While our simulations are limited to amorphous structures and broadened dihedral populations at high temperatures, it appears at first that our sampled conformations are in qualitative agreement with such experimental findings. Furthermore, our data allow for a systematic examination of the impact of composition beyond the perfect meso and racemo sequences. Still, we employ metrics agnostic to any labeling of dihedral populations.

**Figure 8:**
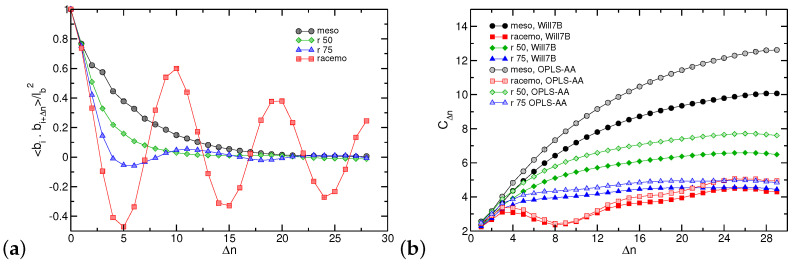
(**a**) Normalized correlation of the C_2_-C_3_ bond vector (with length $l_{b}$) for PNB-N30 and (**b**) characteristic ratio ($C_{\Delta n}=\;<R_{\Delta n}^{2}>/2\left(\Delta n-1\right)l^{2}$, with l=1.53 Å) as a function of monomer distance (Δn) for PNB-N30 at 800 K, from simulations with optimized Williams7B and OPLS-AA.

Figure 8a presents the correlation of the C_2_-C_3_ bond vector along the chain (corresponding to 〈cosθ〉 where θ is the angle between the tangent vectors at monomer *i* and i+Δn). In random coils, this function is used to calculate persistence length since it characterizes correlations between proximal bonds and follows an exponential decay to zero. A clear oscillatory behavior is observed for racemo oligomers, which indicates their affinity to assert a helical conformation. The period appears consistent with ≈10 repeat units, although caution has to be exercised given the relative short chains in the atomistic systems. Given this long period, a small fraction of meso sequence suffices to disrupt such helical arrangements, which are barely for the 75% racemo composition. An intriguing observation is that once a sufficient number of successive meso segments (>50%) are present, the decay appears to extend over longer sequences (higher persistence lengths), suggesting a potential non-monotonic dependence of chain rigidity. Comparing our data to Buono’s study, our helices present a longer period (ten versus six repeat units [53]); this deviation could originate from the high temperatures modeled and the absence of any crystalline packing in our simulations.

The conformation of PNB chains can also be examined by probing the average squared internal separations $\langle R_{\Delta n}^{2}\rangle$ between repeat units that are Δn monomers apart [99,100]. For random coils, $\langle R_{\Delta n}^{2}\rangle$ is expected to increase linearly with Δn. Therefore, normalized to the number of backbone skeletal bonds (multiplied with a squared bond length chosen herein to be $2*1.53^{2}$ Å^2^), data should converge to the characteristic ratio $C_{\infty }$ as Δn→∞. Figure 8b presents such data where it is evident that N=30 is barely long enough to reach a limiting behavior for the meso systems. Results from chains with high racemo compositions are inconclusive due to the helical behavior discussed previously that requires significantly higher molecular weights to be studied. We supplemented our calculations with data using the generic OPLS-AA force field to support our results further. While, as discussed previously, intramolecular repulsions with the Lennard–Jones functional form are overestimated (leading to higher extensions), qualitatively, similar behavior is reproduced as shown in Figure 8b.

### 3.4. Coarse-Grained Model

**Figure 9:**
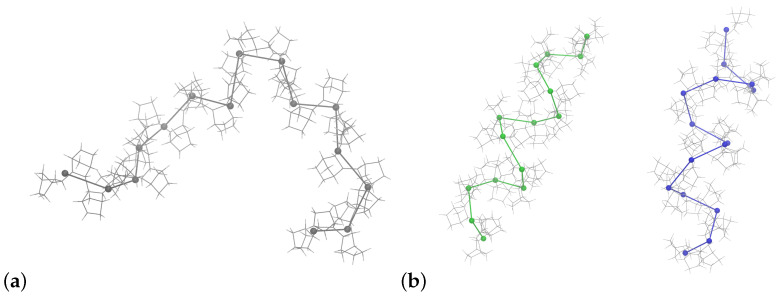
CG mapping scheme based on mapping the center-of-mass of two consecutive monomers to a single CG particle shown for (**a**) meso (dark grey) and (**b**) racemo PNB-30 chains at 800 K. Helical racemo chains can present a left-handed (green) or right-handed (blue) conformation when maintaining a specific end as the start of the chain.

Following our previous results, it is apparent that in addition to accurately representing local interactions and conformations, modeling long PNB chains presents substantial challenges, particularly given their low mobility and high $T_{g}$. Multiscale modeling methods could tackle such spatiotemporal limitations [101,102] but have not yet been proposed for PNBs. Equipped with our extensive simulations of oligomeric compounds, we designed lower-resolution coarse-grained (CG) representations for two of the studied sequences, the 100% meso and 100% racemo chains. We mapped two norbornene monomers to a single CG particle; therefore, intramolecular dihedral interactions imposed in the CG model could directly capture correlations between repeat units that are several monomers apart. We employed the iterative Boltzmann inversion method [103,104,105,106] as we have performed in past studies [107,108]. Figure 9 presents atomistic chains of N=30 mapped to the lower resolution model based on the scheme implemented.

**Figure 10:**
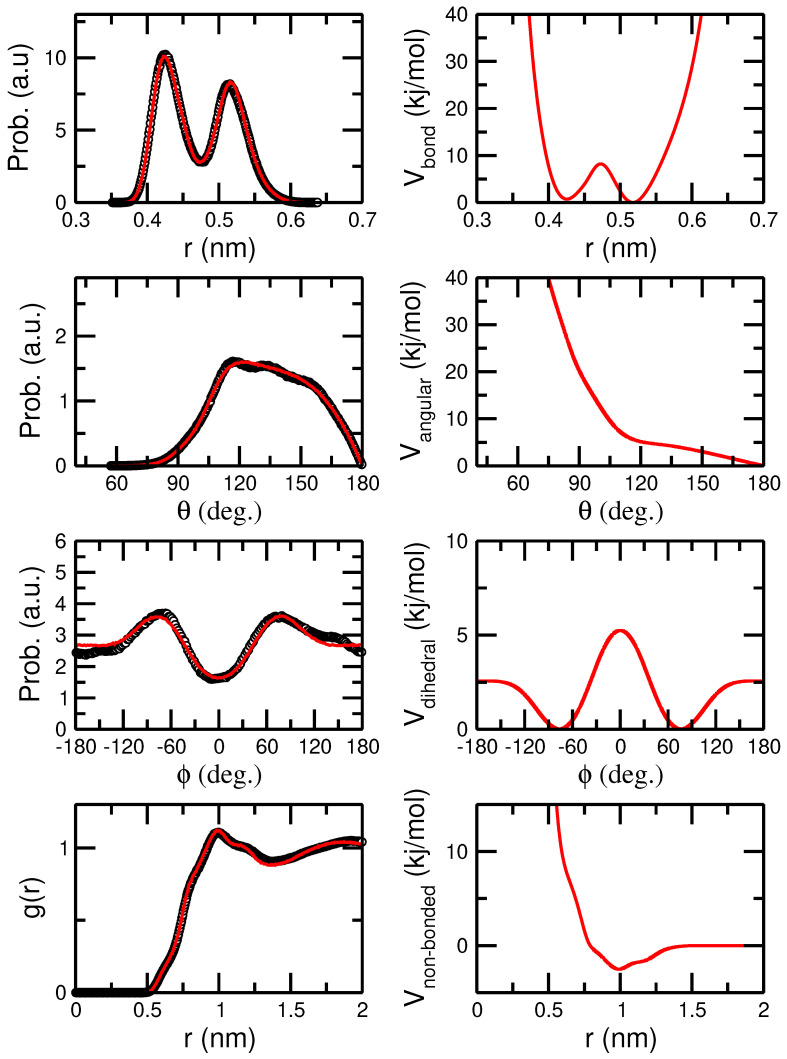
Distributions of bonds, angles, dihedrals, and pair distribution function between CG beads based on a two monomer to single CG particle mapping for the meso N=30 sample at 800 K (black symbols, left column of graphs). Continuous red lines present sampled distributions using the derived force field detailed in the right column.

Figure 10 presents the extracted force field (lines, right column) optimized to match the distribution of bonds, angles, and dihedrals, and the pair distribution function as calculated from the mapped atomistic trajectory with N=30, 800 K (CG particles that interact via intramolecular terms are excluded from non-bonded interactions and intramolecular distributions were calculated excluding the three repeat units proximal to chain ends). In all cases, the predicted data by the CG force field (lines, left column) are in good agreement with the mapped distributions from the atomistic trajectories (open symbols, left column).

**Figure 11:**
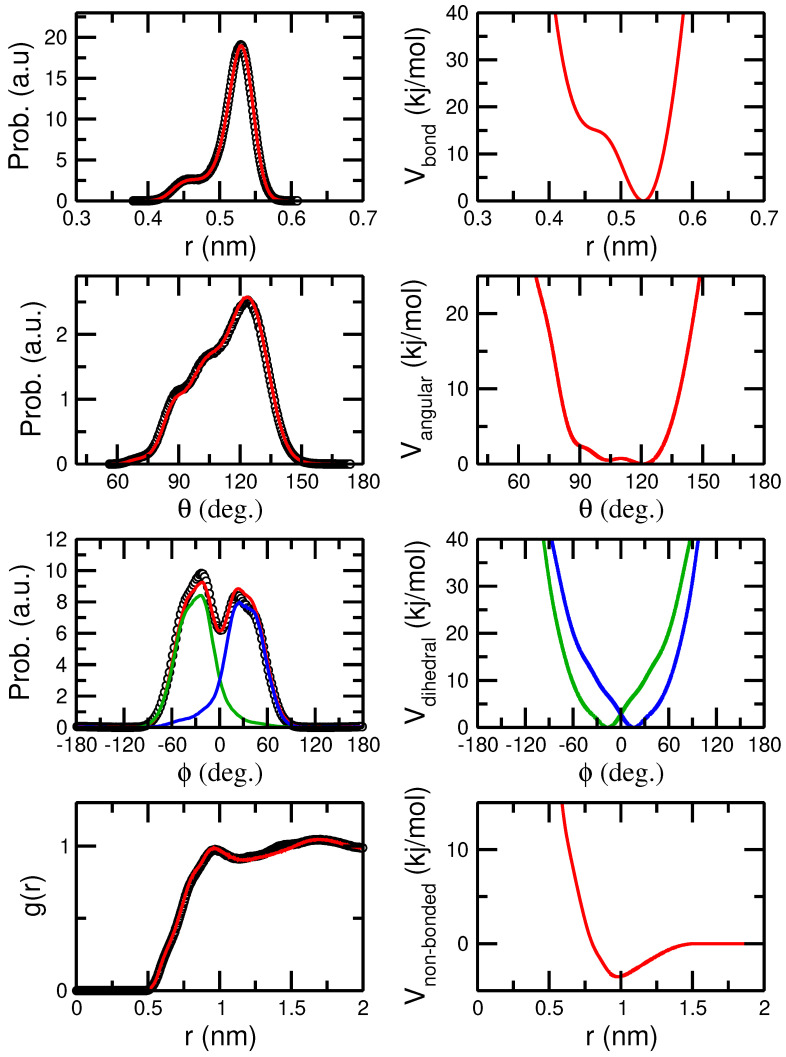
Distributions of bonds, angles, dihedrals, and pair distribution function between CG beads based on a two monomer to single CG particle mapping for the racemo N=30 sample at 800 K (black symbols, left column of graphs). Continuous red lines present sampled distributions using the derived force field detailed in the right column.

Moving to 100% racemo sequences, we observed that our oligomeric chains presented extensive correlations across the whole molecule. This is best illustrated by the dihedral distribution of the mapped trajectories presented in the left column of Figure 11. While both negative and positive populations are probed, dihedrals in individual chains are highly correlated. Grouping chains with an average negative value resulted in an unimodal distribution corresponding to left-handed helices (see also Figure 9) while the opposite corresponds to right-handed helices with most dihedrals in the sequence being positive. To capture this behavior, we derived a dihedral potential targeting the distribution centered at negative values and, once optimized, modeled a mixture of 50:50 left-handed and right-handed helices by mirroring the potential around the ϕ= 0° value. Simulations with this composition resulted in total dihedral distributions that captured the total atomistic populations (see red line, Figure 11). Note, that occasionally positive values are observed even in left-handed helices, and the same is true for right-handed conformations, but during simulations with the CG model chains, they remain largely helical by design.

**Figure 12:**
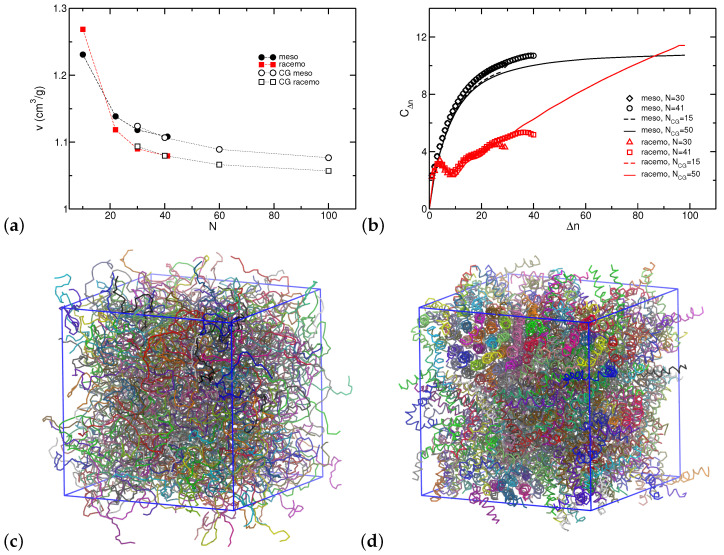
(**a**) Specific volume (*v*) of PNB as a function of chain length for AA and CG simulations at 800 K. (**b**) Internal separations cast as characteristic ratio $C_{\Delta n}$ at 800 K, as calculated from AA (open symbols) and coarse-grained (lines) simulations. Snapshot of a CG representation of the longest system examined (N=100, $N_{\mathrm{CG}}=50$) at 800 K for the (**c**) meso and (**d**) racemo architecture. Chains in the systems are depicted as lines in different colors. Note the small transient crystalline nucleus at the top left corner of the racemo system.

Simulations with the CG model enable us to examine the dependence of properties on the degree of polymerization. We modeled chains composed of 15, 20, 30, and 50 particles corresponding to atomistic systems with twice the number of repeat units (30, 40, 60, and 100). Figure 12 presents data on the variation of the specific volume *v*; the CG model is in excellent agreement with atomistic results and captures the anticipated decrease in the specific volume with the dilution of chain ends [93,100]. Proceeding to conformational characteristics of the lower resolution model we find that spatial arrangements for the 100% meso sequences are captured reasonably well as shown in Figure 12b with a plateau indicating random coil behavior for molecules longer than 50 repeat units.

As expected by atomistic simulations and design of CG model, the 100% racemo chains exhibit helical behavior with a pitch of ≈10 repeat units. Given the rigid tubular molecules, at long length scales, squared separations $<R_{\Delta n}^{2}>$ increase with the square of the topological distance (rigid rod scaling), resulting in a characteristic ratio that grows linearly with Δn. Thus, the model predicts that while low molecular weight racemo chains can display a more compact conformation, eventually, chain sizes exceed those of 100% meso chains with the same degree of polymerization.

The question remains whether longer racemo sequences can exert organization to crystalline forms. We probed our trajectories and occasionally detected transient nuclei that appeared and dissolved in time but did not persist over trajectories exceeding 100 ns at 800 K. This is further illustrated in Figure 12c,d presenting visual representations of model $N_{CG}=50$ systems. Potentially at lower temperatures, a transition to crystalline forms could be present. However, caution has to be exercised given that we anticipate that our CG potentials are state-dependent and would need reparametrization as often required in applications of multiscale modeling [99,109]. Therefore, future multiscale studies will focus on the impact of temperature as well as substitutions in the PNB repeat units that are the subject of molecular design in applications with these polymers.

## 4. Discussion

We presented a systematic multiscale computational study to directly examine the effect of stereochemistry on the properties of unsubstituted polynorbornenes. We initially reviewed the ability of several all-atom force fields to capture calculations with density functional theory using model dimers and trimers with different stereochemistry. Subsequently, given the superior performance of Williams7B, we further optimized the backbone dihedral potentials to better align with DFT rotational energy barriers. Molecular dynamics simulations in the melt state (800 K) suggest that racemo chains form tubular helical conformations with a pitch of 10 repeat units in contrast to meso systems that display rigid and extended chains ($C_{\infty }\approx 11$) with no clear evidence of helicity. All systems present high glass transition temperatures (550–600 K); however, racemo samples appear to exhibit higher cohesive energies as evidenced both by a slightly higher $T_{g}$ and densities in the melt state. A small fraction of meso sequences is sufficient to perturb helicity in high-racemo chains, and the rigidity of atactic chains composed of a random sequence of racemo and meso dyads is potentially a non-monotonic function of composition. In addition, we proposed a CG representation that suffices to capture the long-range conformational characteristics of PNB chains with 100% meso or 100% racemo tacticity, hinting at potential crystallization of the latter at higher molecular weights and lower temperatures.

Selective functional substitution of vinyl-addition polynorbornene presents significant opportunities for the molecular design of polymers with unique properties. The multiscale scheme detailed in our study provides a framework to model high-performance polymers at high degrees of polymerization by overcoming equilibration challenges present with these rigid chemical architectures. Backmapping procedures [107,108,110] could be utilized to reintroduce atomistic details to equilibrated coarse-grained structures for addressing applications such as gas separation membranes where atomistic details are deemed necessary [6,7,8,9,10,11,12,13].

## Data Availability

The original contributions presented in the study are included in the article. Further inquiries can be directed to the corresponding authors.

## Abbreviations

The following abbreviations are used in this manuscript:

AA	All-atom
CG	Coarse-grained
DFT	Density functional theory
MD	Molecular dynamics
PNB	Vinyl-addition polynorbornene
RIS	Rotational isomeric state
$T_{g}$	Glass transition temperature
